# Molecular Basis of the Inhibition of Voltage-Gated Potassium Channel Kv1.1 by Chinese Tarantula Peptide Huwentoxin-XI

**DOI:** 10.3390/toxins18030124

**Published:** 2026-03-01

**Authors:** Xuan Luo, Yuan Yin, Fenghua Wang, Xinyu Li, Shujun Wang, Yumei Yang, Chunbing Zheng, Jing Liu, Meichun Deng

**Affiliations:** 1 Hunan Province Key Laboratory of Basic and Applied Hematology, Department of Biochemistry and Molecular Biology, School of Life Sciences, Central South University, Changsha 410013, China; 2 Yuanpin Cell Biotechnology Group Co., Ltd., Changsha 410100, China; 3 College of Physical Education, Xinjiang Normal University, Urumqi 830054, China; 4 Xiangya School of Medicine, Central South University, Changsha 410013, China; 5 Changsha Institute of Industrial Technology for Stem Cell and Regenerative Medicine, Changsha 410100, China; 6 Hunan Key Laboratory of Animal Models for Human Diseases, Hunan Key Laboratory of Medical Genetics, School of Life Sciences, Central South University, Changsha 410013, China

**Keywords:** tarantula toxin, potassium channels, Kv1.1, patch-clamp, pain

## Abstract

Huwentoxin-XI (HWTX-XI) is a 55-amino acid peptide belonging to the family of spider Kuntiz-type toxins (KTTs), isolated from the venom of the Chinese tarantula *Cyriopagopus schmidti*. Under whole-cell voltage-clamp conditions, HWTX-XI was found to block Kv1.1 potassium channels but had no effect on other potassium channel subunits (Kv1.4, Kv2.1, Kv3.1 and Kv4.2), sodium channels or calcium channels. In the present study, it was found that the substitution of Tyr379 by the valine in the filter region significantly decreased the affinity of toxin HWTX-XI by about 90-fold, indicating that the Kv1.1 filter region is a critical determinant of HWTX-XI potassium channel activity. After intrathecal or intraplantar injections, HWTX-XI decreased the mechanical nociceptive threshold (hyperalgesia) for a long-lasting period. HWTX-XI also significantly increased the firing frequency in mouse DRG neurons. The novel function of HWTX-XI makes it a new tool for studying the relationship between spider toxins and Kv1.1 channels and suggests that Kv1.1 channels might be a novel potential target for preventing and/or treating neuropathic pain.

## 1. Introduction

Voltage-gated potassium (Kv) channels, which are tetrameric plasma membrane proteins, represent one of the largest and most evolutionarily conserved ion channel families, with expression found in both excitable and non-excitable tissues [1]. As critical regulators of neuronal excitability, Kv1.1 potassium channels (encoded by KCNA1) are widely expressed throughout the central and peripheral nervous systems. Pathogenic alterations in these channels are known to cause neurological conditions, including episodic ataxia type 1 and epilepsy [2,3]. A key mechanoregulatory role of Kv1.1 is to attenuate signal transduction in sensory fibers, thereby shaping the perception of both tactile and painful mechanical stimuli [4]. Expression of the KCNA2-encoded Kv1.2 channel is most prominent in the central nervous system. As such, it has become an essential structural template for investigating the function of voltage-gated ion channels [5]. Kv1.3 channels (encoded by KCNA3) have emerged as a therapeutic target in autoimmune conditions, including multiple sclerosis. This is due to their pivotal role in modulating the immune responses of chronically activated effector memory T cells [6,7]. Crystallographic studies of the Kv1.2 potassium channel and its Kv1.2–Kv2.1 chimera have elucidated the architecture of the Shaker channel family, which is characterized by six transmembrane segments (S1–S6) and a central pore region. Architecturally, the pore region comprises the S5 and S6 segments and the intervening pore loop, which includes the turret, pore helix, selectivity filter, and pre-S6 loop. This structure assembles from four similar or identical pore-forming α-subunits [8,9,10]. Thus, the broad expression of Kv1 potassium channels in mammalian tissues underpins their involvement in a wide spectrum of biological processes, spanning the control of neuronal excitability to the maturation of T-lymphocytes [11].

Studies utilizing toxin peptides from scorpions, sea anemones, snakes, and cone snails to block Kv1 channel currents (Kv1.1–Kv1.7) have been instrumental in elucidating the structure–function relationships of potassium channels [12,13,14,15,16]. The study of inhibitory peptide toxins offers a valuable approach to investigating the molecular basis of multifaceted protein–protein interactions in potassium channels and has yielded key insights into the structure of their pore-forming regions [17,18,19]. Beyond their inhibitory function, these peptide toxins are invaluable tools for dissecting the specific contributions of potassium channels to ionic currents. For instance, scorpion-derived toxins such as maurotoxin, margatoxin, and hongotoxin display varied affinities toward different Kv1 channels, helping to delineate specific channel functions [20,21]. BgK is a peptidic inhibitor of Kv1.1, Kv1.2, and Kv1.3 potassium channels, originally isolated from the venom of the sea anemone *Bunodosoma granulifera* [22]. Dendrotoxins (DTX-I, DTX-K, α-DTX, and δ-DTX) are 57–60-amino acid Kv1 channel blockers containing three disulfide bonds, isolated from the venoms of *Dendroaspis polylepis* and *Dendroaspis angusticeps*. Spider venom peptide toxins, conversely, have been invaluable tools for probing Kv4 and Kv2 channels [23,24,25,26]; however, few spider toxins are found to bind to Kv1 channels.

Huwentoxin-XI (HWTX-XI) is structurally homologous with dendrotoxins, which is a specific Kv1.1 channel blocker from the venom of the Chinese tarantula *Cyriopagopus schmidti* [27]. It is also known that HWTX-XI shares high structural homology with these dendrotoxins, suggesting a possible similar function. However, it remains unverified whether HWTX-XI indeed inhibits Kv1.1 channels with high affinity and, if so, whether its mechanism involves the same pore region as classical dendrotoxins. Furthermore, the functional consequences of such interaction—particularly in the context of neuronal excitability and pain signaling—have not been experimentally demonstrated. To test the hypothesis that the turret and filter regions form the primary interaction sites in the outer vestibule, we employed HWTX-XI as a molecular probe to pinpoint the specific residues on Kv1 channels that confer its binding selectivity for Kv1.1 versus Kv1.2 and Kv1.3. We also determined the effects of HWTX-XI on the mechanical hypersensitivity of mice and dorsal root ganglion (DRG) neuronal excitability. These findings broaden our mechanistic insight into spider toxin-Kv1 channel interactions.

## 2. Results

### 2.1. Gene Expression, Purification and Identification of HWTX-XI

**Figure 1:**
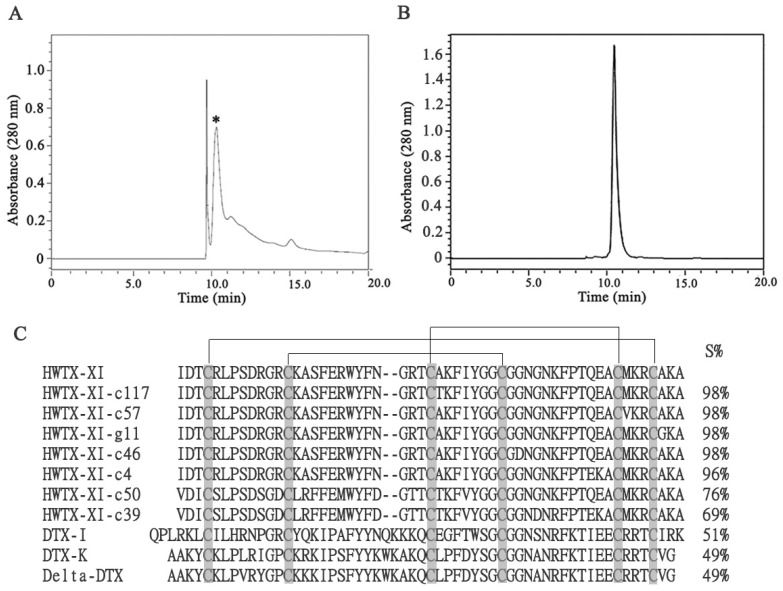
Purification and sequence alignment of HWTX-XI. (**A**) Preparative RP-HPLC chromatogram of the HWTX-XI fraction on a C18 column. The asterisk denotes the target fraction. (**B**) Analytical RP-HPLC profile of the purified peptide on a C18 column, demonstrating its homogeneity. For both (**A**,**B**), a linear gradient of 20–45% acetonitrile (containing 0.1% TFA) was applied over 30 min at a flow rate of 3.0 mL/min. (**C**) Sequence alignment of HWTX-IV with other related toxin peptides from animal venoms. HWTX-XI-c117, HWTX-XI-c57, HWTX-XI-g11, HWTX-XI-c46, HWTX-XI-c4, HWTX-XI-c50, and HWTX-XI-c39 are from the Chinese tarantula *Cyriopagopus schmidti*. DTX-I and DTX-K are from the venom of black mamba snake *Dendroaspis polylepsis*. Delta-DTX is from snake *Dendroaspis angusticeps*. The diagram illustrates the disulfide bridging pattern (I–VI, II–IV, and III–V) above the sequence alignment. The cysteine residue is located at the position indicated by the shaded area. The percentage similarity (S%) is shown to the right of the sequences.

The recombinant peptide HWTX-XI was successfully expressed in *Saccharomyces cerevisiae* and purified from the culture supernatant using cation-exchange chromatography followed by reverse-phase HPLC (Figure 1A). Peptide purity was then assessed by analytical RP-HPLC on a C18 column, using an acetonitrile gradient in 0.1% trifluoroacetic acid. RP-HPLC analysis revealed a single symmetrical peak for HWTX-XI, indicating high homogeneity (Figure 1B). Its molecular mass was determined by MALDI-TOF MS to be 6166.36 Da, which is 6 Da lower than the calculated mass of 6172.2 Da for the reduced form. This mass difference corresponds to the formation of three intramolecular disulfide bonds, confirming that all six cysteine residues are engaged in disulfide bridge formation.

Alignment with other animal toxins shows that HWTX-XI shares significant similarity with HWTXs (HWTX-XI-c117, 98%; HWTX-XI-c57, 98%; HWTX-XI-g11, 98%; HWTX-XI-c46, 98%; HWTX-XI-c4, 96%; HWTX-XI-c50, 76%; and HWTX-XI-c39, 69%) from venom of Chinese tarantula *Cyriopagopus schmidti*; DTX-I (51%) and DTX-K (49%), potassium channel blockers from black mamba snake *Dendroaspis polylepsis* venom; and Delta-DTX (49%), a potassium channel blocker from snake *Dendroaspis angusticeps*.

### 2.2. Biological Activities of the Recombinant HWTX-XI

**Figure 2:**
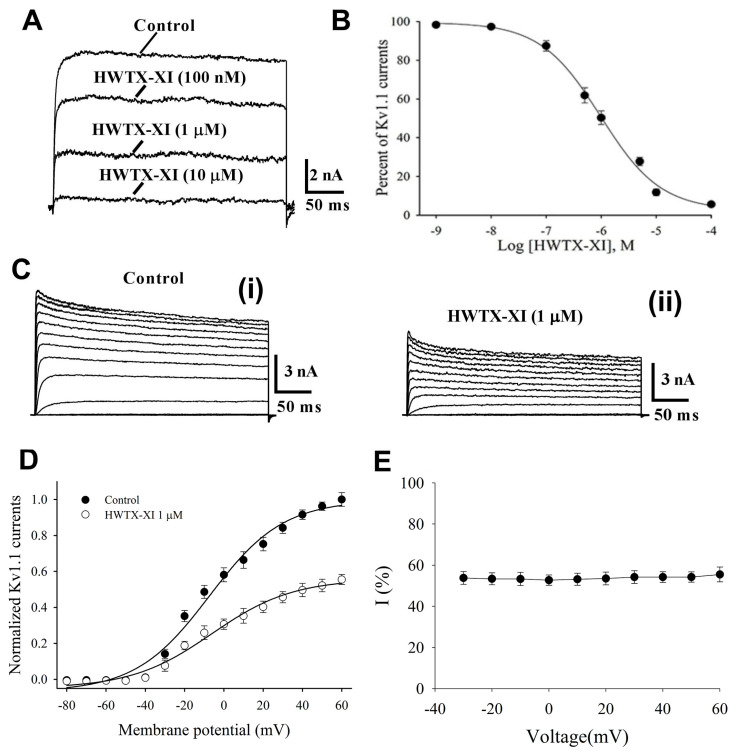
HWTX-XI inhibits Kv1.1 channels. (**A**) Current traces from HEK 293T cells expressing Kv1.1, demonstrating block by HWTX-XI. The voltage protocol (inset) was a 300 ms step to +20 mV from −80 mV. (**B**) Dose‒response curve of the inhibition. Each point represents the mean ± S.E. from 6 to 8 independent cells. The curve is a fit to the Hill equation (Equation (1)) (see “Materials and methods”). (**C**) Superimposed Kv1.1 current families elicited by voltage steps (−80 to +60 mV) before and after applying 1 μM HWTX-XI. (**D**) Steady-state I–V relationships derived from the experiment shown in (**C**), summarizing data from 8 cells (mean ± S.E.M.). (**E**) The percentage of unblocked current in the presence of the toxin plotted against test potential, demonstrating voltage-independent inhibition (mean ± S.E.M.).

In order to detect the biological activities of HWTX-XI, the electrophysiological effects of the toxin were determined on the Kv1.1 channels expressed on HEK293T cells using the whole-cell patch-clamp technique. To assess its activity, the effect of HWTX-XI (1 nM–100 μM) on Kv1.1 channels was measured under voltage clamp. Outward potassium currents, evoked by a 300 ms step to +20 mV from a −80 mV holding potential, were inhibited by the peptide in a concentration-dependent manner. HWTX-XI at 0.1 and 1 μM significantly inhibited Kv1.1 currents on HEK293T cells. After exposure to 10 μM HWTX-XI, almost no residual current was detected (Figure 2A). Figure 2B presents the dose–response curves fitted with the Hill equation (see Materials and Methods, Equation (1)), yielding an IC_50_ value of 0.97 μM and a Hill coefficient (nH) of 0.98 for Kv1.1 channel inhibition. A Hill coefficient near 1 suggests a 1:1 binding stoichiometry between HWTX-XI and the channel. This is identical to previous work [27]. Our above data illustrates that rHWTX-XI might display similar biological activities with natural HWTX-XI on Kv1.1 potassium channels.

The voltage dependence of HWTX-XI action on Kv1.1 was assessed using a series of depolarizing steps (−80 to +60 mV). The I–V relationships (Figure 2C,D) show that 1 μM HWTX-XI inhibited currents across all voltages tested after 3 min of application but did not shift the activation threshold. The degree of inhibition was consistent between −30 and +60 mV (Figure 2C–E), indicating voltage-independent blockade. Moreover, the toxin’s inhibition was both rapid and reversible [28]. The rapid onset and offset of the HWTX-XI action indicates that the toxin might bind to the extracellular site of Kv1.1 channels.

### 2.3. Effects of HWTX-XI on Voltage-Gated Potassium Channels, Sodium Channels, and Calcium Channels

**Figure 3:**
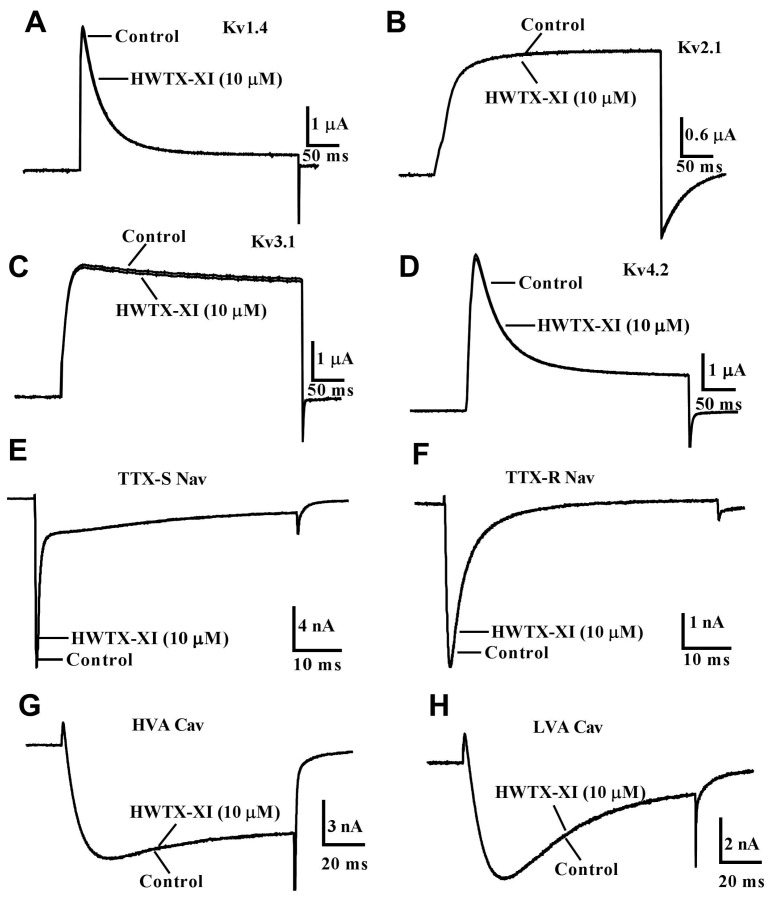
Selectivity of HWTX-XI for ion channels. (**A**–**D**) An amount of 10 µM of HWTX-XI showed no detectable effect on Kv1.4 (**A**), Kv2.1 (**B**), Kv3.1 (**C**), or Kv4.2 (**D**) potassium channels expressed in *Xenopus laevis* oocytes. Currents were evoked by a 300 ms step to +20 mV from −90 mV. (**E**,**F**) The toxin (10 µM) did not affect TTX-sensitive (**E**) or TTX-resistant (**F**) voltage-gated sodium channels in mouse DRG neurons. Sodium currents were elicited by a 50 ms depolarization to −10 mV from −80 mV. (**G**,**H**) Similarly, 10 µM of HWTX-XI had no obvious effect on high-voltage-activated (**G**) or low-voltage-activated (**H**) calcium channels. HVA and LVA currents were evoked by 100 ms steps to 0 mV (from −40 mV) and −50 mV (from −90 mV), respectively.

In our previous work, we found that HWTX-XI had less effect on Kv1.2 and Kv1.3 channels in comparison to Kv1.1 channels [28]. In this study, we tested HWTX-XI on four potassium channel isoforms (Kv1.4, Kv2.1, Kv3.1, and Kv4.2) expressed in *Xenopus laevis* oocytes. Using a depolarizing step from –90 to 0 mV, outward potassium currents were recorded. Bath application of 10 µM HWTX-XI had no discernible effect on current amplitudes (Figure 3A–D). Next, to assess selectivity, we tested the toxin on voltage-gated sodium and calcium channels in mouse dorsal root ganglion (DRG) neurons. At 10 µM, HWTX-XI did not affect either TTX-sensitive or TTX-resistant sodium currents from mouse DRG neurons (Figure 3E,F). Similarly, the toxin had no significant effect on high- or low-voltage-activated calcium currents under whole-cell voltage clamp (Figure 3G,H).

### 2.4. The Filter Region but Not the Channel Turret of Kv1.1 Channels Determines HWTX-XI Sensitivity

**Figure 4:**
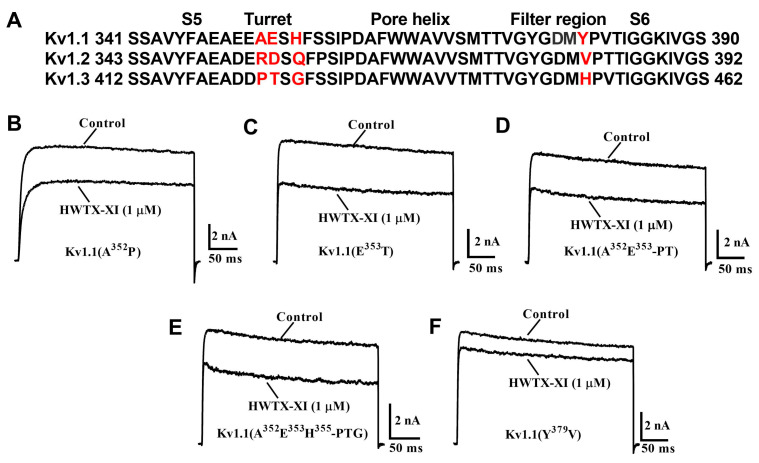
Comparative analysis of HWTX-XI binding to Kv1.1 channel mutants. (**A**) Comparative alignment of the pore regions from Kv1.1, Kv1.2, and Kv1.3. Residues that are not identical across the three channels are denoted in red. B-G, Current traces in the absence (control) or presence of HWTX-XI on Kv1.1-A352P (**B**), Kv1.1-E353T (**C**), Kv1.1-A352P/E353T (**D**), Kv1.1-A352P/E353T/H355G (**E**), and Kv1.1-Y379V (**F**) mutant channels. Currents were recorded in response to a 300 ms depolarization to +20 mV from −80 mV.

The filter region and the channel turret are the two primary domains in Kv1.x channels responsible for inhibitory peptide recognition. Specifically, they have been identified as key determinants mediating binding and blockade by peptide toxins such as α-dendrotoxin (from snake venom) and BgK (from sea anemone venom) [29]. We therefore asked whether the filter region and channel turret are also critical for HWTX-XI binding and inhibition of Kv1.1 channels. Sequence alignment of the turret and filter domains of Kv1.1, Kv1.2, and Kv1.3 revealed a high degree of conservation (>90% identity) in the pore region. Differences were confined to only four positions in the turret and near the selectivity filter (Figure 4A). We performed site-directed mutagenesis to replace these four Kv1.1 residues with the corresponding amino acids found in Kv1.2 or Kv1.3. All together, we constructed six channel mutants (Kv1.1-A^352^P, Kv1.1-E^353^T, Kv1.1-A^352^E^353^/PT, Kv1.1-H^355^Q, Kv1.1-A^352^E^353^H^355^/PTG, and Kv1.1-Y^379^V). HEK293T cells were transfected with expression vectors for wild-type or mutant Kv1.1 channels. Following transfection, potassium channel activity was measured in the presence of HWTX-XI to assess the inhibitor sensitivity of each construct.

**Figure 5:**
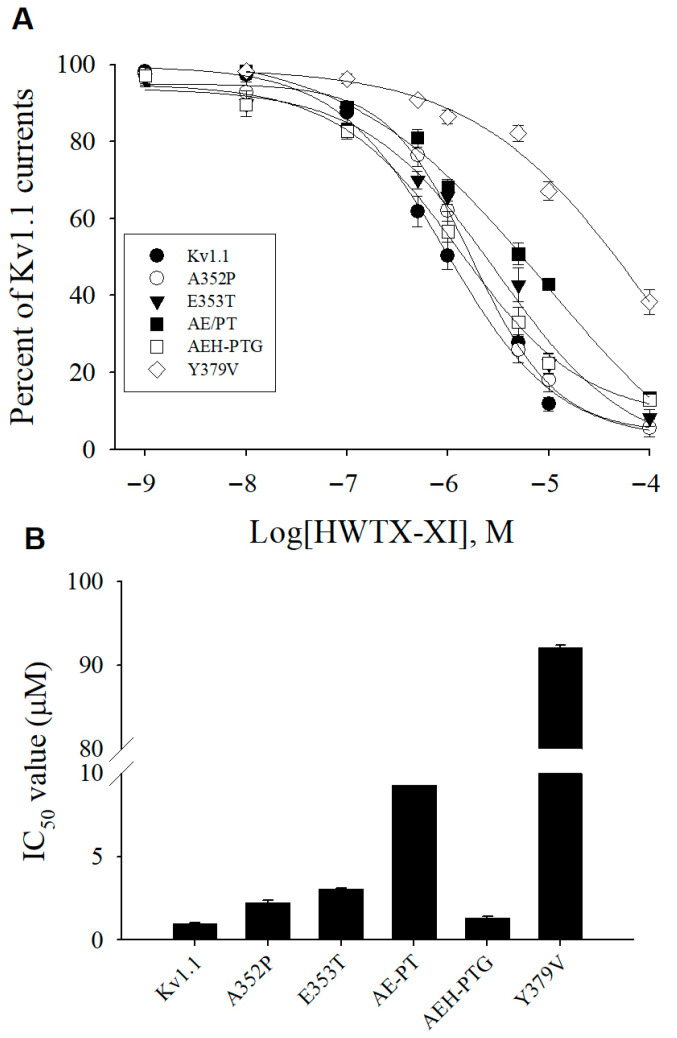
Concentration dependence on the inhibition of Kv1.1 and Kv1.1 mutants by HWTX-XI. (**A**). Normalized current inhibition by various concentrations of HWTX-XI on Kv1.1, Kv1.1-A352P, Kv1.1-E353T, Kv1.1-H355Q, Kv1.1-A352P/E353T, Kv1.1-A352P/E353T/H355G, and Kv1.1-Y379H channels. Every data point (mean ± S.E.) comes from 6 to 8 separated experimental cells. (**B**) IC_50_ values for HWTX-XI on Kv1.1, Kv1.1-A352P, Kv1.1-E353T, Kv1.1-H355Q, Kv1.1-A352P/E353T, Kv1.1-A352P/E353T/H355G, and Kv1.1-Y379H channels. Data are shown as means ± SEM.

Potassium currents from mutant channels were recorded in whole-cell configuration using 300 ms test pulses to +20 mV from a holding potential of −80 mV (0.2 Hz). These experiments revealed that substituting the two turret residues (A352 and E353) markedly decreased the toxin’s binding affinity. Following application of 1 μM rHWTX-XI, the current amplitude through mutant Kv1.1 channels A^352^P and E^353^T was only depressed by 37.9 ± 2.6% and 34.3 ± 2.1%, respectively (Figure 4B,C). Inhibition of the double mutant Kv1.1-A^352^E^353^/PT currents by 1 μM rHWTX-XI was a little lower (28.7 ± 1.9%, Figure 4D). However, when the residue H355 in the channel turret was replaced with Gln, the sensitivity of the channel to HWTX-XI was greatly increased and 47.5 ± 4.1% of the H^355^Q mutant channel could be inhibited by 100 nM HWTX-XI (Figure 4E). Interestingly, 1 μM HWTX-XI inhibited triple mutant Kv1.1-A^352^E^353^H^355^/PTG currents by 43.4 ± 5.3% (Figure 4F), which is close to the inhibition of WT Kv1.1 currents. The dose–response relationships in Figure 5, when fitted with the Hill equation, yielded IC_50_ values of 2.25, 3.06, 9.25, 0.12 and 1.31 μM on A^352^P, E^353^T, A^352^E^353^/PT, Kv1.1-H^355^Q, and A^352^E^353^H^355^/PTG mutant Kv1.1 channels, respectively. The IC_50_ value for triple mutant Kv1.1- A^352^E^353^H^355^/PTG was close to the value for WT Kv1.1 channels (Figure 5). These data demonstrate that the residues in the channel turret are not a critical determinant of HWTX-XI on potassium channel selectivity and imply that the potassium channel filter region may be the candidate domain.

To confirm the role of the residue of filter region in HWTX-XI inhibition, we investigated the effects of HWTX-XI on the channel mutant Kv1.1-Y^379^V. After exposed to 1 μM HWTX-XI, 86.4 ± 1.7% of the Y^379^V mutant channel current remained available for activation (Figure 4F). As 1 μM HWTX-XI only inhibits 13.6% of the Kv1.1-Y^379^V current, the IC_50_ value can be roughly estimated as 92.1 μM (Figure 5), showing that the Y^379^V mutation reduces toxin sensitivity by at least 90-fold. These data indicate that the Kv1.1 filter region is the critical determinant for HWTX-XI potassium channel activity. These data provide unequivocal evidence that the Kv1.1 filter region is essential for HWTX-XI potassium channel selectivity and activity.

### 2.5. Effects of HWTX-XI on Mechanical and Thermal Hypersensitivity

**Figure 6:**
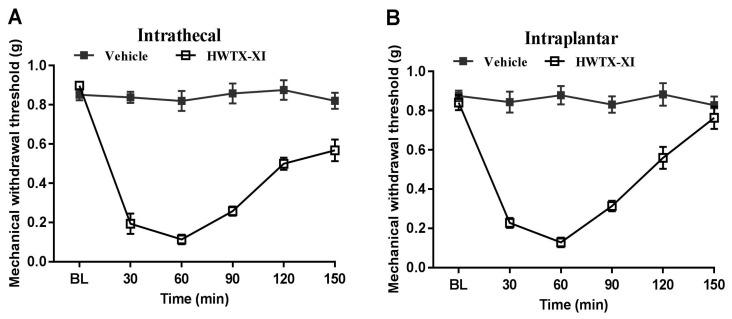
Effects of HWTX-XI on mechanical sensitivity. (**A**,**B**) Mechanical hypersensitivity assessed using the von Frey test following intrathecal (**A**) or intraplantar (**B**) administration of HWTX-XI. The data are presented as the mean ± SEM.

Clinical observations indicate that human envenomation by *Cyriopagopus schmidtiis* is notable for intense pain and significant local swelling [30]. Having known the importance of Kv1.1 in the pain transmission, we next determined whether HWTX-XI also induces mechanical and thermal hypersensitivity. We administered intrathecal or intraplantar HWTX-XI to mice and then examined the withdrawal thresholds in response to noxious mechanical and heat stimuli. Intrathecal or intraplantar injection of HWTX-XI (0.6 nmol) caused a significant decrease in the baseline tactile withdrawal thresholds in mice (*n* = 8, Figure 6A,B). HWTX-XI treatment produced a prolonged reduction in paw withdrawal thresholds that persisted for at least 150 min after administration, without inducing spontaneous nocifensive behaviors (*n* = 7). In contrast, vehicle treatment had no effect on mechanical thresholds (Figure 6A,B).

### 2.6. Effects of HWTX-XI on Action Potentials in Mouse DRG Neurons

**Figure 7:**
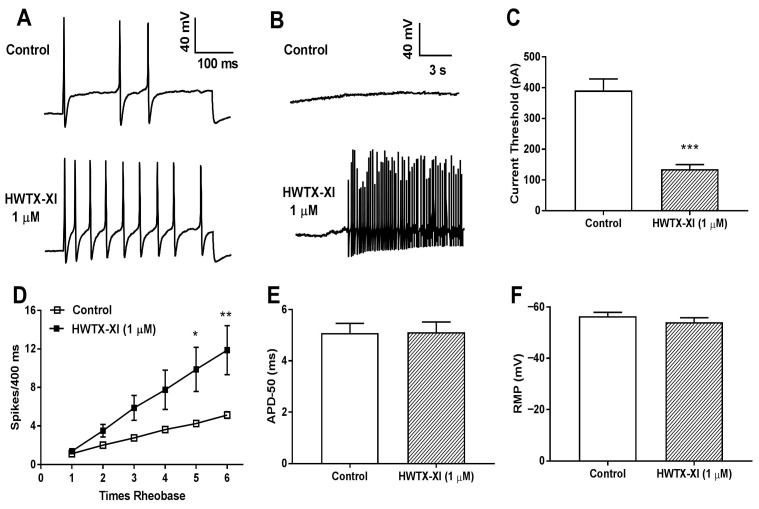
HWTX-XI modulates the firing properties of rat dorsal root ganglion (DRG) neurons. (**A**) Representative current-clamp traces from a DRG neuron before (control) and after bath application of 1 µM HWTX-XI. The membrane potential was held at −60 mV via steady hyperpolarizing current injection. Spontaneous firing activity of a DRG neuron recorded before and after applying 1 µM HWTX-XI, with the membrane potential maintained at −60 mV. (**B**) Some DRG neurons exhibited ectopic spontaneous activity (action potentials) after treatment with 1 μM HWTX-XI. (**C**–**F**) Quantitative analysis of the effects of 1 µM HWTX-XI on (**C**) current threshold for action potential initiation, (**D**) firing frequency, (**E**) action potential duration at 50% repolarization (APD50), and (**F**) resting membrane potential (RMP). Data are shown as means ± SEM. * *p* < 0.05, ** *p* < 0.01, *** *p* < 0.001 vs. the control group.

In the previous study, HWTX-XI was found to evidently reduce the potassium channel currents in mouse DRG neurons [28]. The contribution of HWTX-XI-sensitive Kv currents to DRG neuron firing properties was evaluated by comparing spike activity induced by current injection before and after Kv current blockade with 1 µM HWTX-XI. Application of 1 μM HWTX-XI significantly increased the firing frequency in mouse DRG neurons (Figure 7A,D). Our work also showed that some mouse DRG neurons exhibited ectopic spontaneous activity (action potentials) after treatment with 1 μM HWTX-XI (Figure 7B). We found that application of 1 µM HWTX-XI significantly reduced the current injection threshold required to evoke firing in mouse DRG neurons (Figure 7C). Additionally, HWTX-XI had no significant effect on action potential duration, measured at 50% spike height (Figure 7D); in this regard, its effect resembled that of 4-AP, which also did not alter spike width. Finally, HWTX-XI did not significantly affect the resting membrane potential of these neurons (Figure 7E).

## 3. Discussion

**Figure 8:**
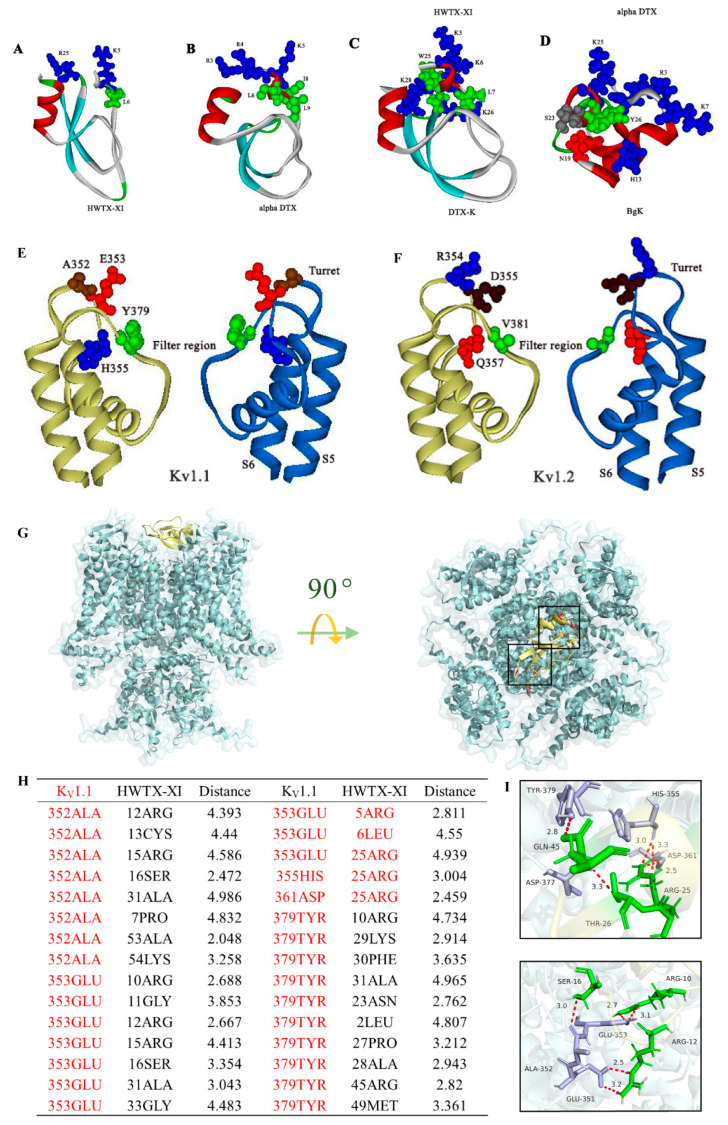
Structural comparison of Kv1 channel toxins and the S5-S6 region of the Kv1.1 and Kv1.2 channels. (**A**,**B**) Models for the S5-S6 region of Kv1.1 (**A**) and Kv1.2 (**B**). Kv1.1 in the S5-S6 region was modeled according to the crystal structure of Kv1.2 (PDB: 1ZWI) from the PDB by using SWISS-MODEL [47]. Filter and turret regions indicated the possible toxin-interacting surfaces. The residues important for Kv1.1 channel binding to HWTX-XI and the similar positions of the Kv1.2 channel are colored. (**C**–**F**) Ribbon diagram of the solution structures of HWTX-XI (**C**, PDB code: 2JOT), alpha DTX (**D**, PDB code: 1DEN), DTX-K (**E**, PDB code: 1DTK), and BgK (**F**, PDB code: 1BGK). The residues important for toxins binding to the Kv1.1 channel are colored. (**G**) Overall structure of the HWTX-XI/Kv1.1 complex. Two orthogonal views of the complex are shown, related to a 90° rotation as indicated by the arrow. HWTX-XI is depicted as a ribbon or cartoon model, and the Kv1.1 channel is shown as a molecular surface. The yellow regions represent HWTX-XI, the cyan regions represent Kv1.1, and the red lines indicate hydrogen bonds.The key hydrogen bonding interaction sites between the two are boxed in black. (**H**) Table listing key intermolecular residue pairs between Kv1.1 and HWTX-XI and their corresponding atomic distances (Å). Data highlight the closest contacts involving critical residues. The key interacting amino acid residues are highlighted in red, with their distances labeled. (**I**) Detailed view of the interaction interface. Specific molecular interactions, namely hydrogen bonds between the key residues of HWTX-XI and Kv1.1, are shown. This panel validates the proximity data from (**H**) and illustrates the nature of the binding forces.

As is well known, elucidating how specific ligands bind to subsets of closely related receptors can greatly accelerate the development of drugs with desired selectivity. There are few spider toxins found to block Kv1 channels; our current understanding of the relationship between Kv1 channels and spider toxins remains fragmented. In the present study, we found that the spider toxin HWTX-XI not only blocked Kv1.1 channels by binding to the filter region of the channels but also caused mechanical hypersensitivity by targeting Kv1.1 channels to induce hyperexcitability of DRG neurons. The extracellular pore entryway, comprising the turret and filter regions, is a critical domain that mediates channel inactivation, animal toxin recognition, and intersubunit interactions [31,32]. The possibility that both the channel turret and the filter region contributed to the high selectivity of HWTX-XI for Kv1.1 over Kv1.2 and Kv1.3 (Figure 8A,B) has been examined by swapping the amino acid residues between the sensitive Kv1.1 and insensitive Kv1.2 or Kv1.3 channels; however, the channel turret had a negligible effect on HWTX-XI selectivity, which was expected because the similar turret region between mutant and wild Kv1.1 channels led to fewer differences in HWTX-XI affinity.

A Tyr above the filter region of voltage-gated potassium channels (Tyr379 in Kv1.1) has been shown to lie at the external mouth of the channel, affecting single-channel rectification and TEA binding [33]. Regarding why a tyrosine (Y) at this position confers high affinity to both a small molecule (TEA) and a large peptide toxin (HWTX-XI), we hypothesize that Y379 may play a dual role in ligand recognition through its aromatic side chain, which can engage in different types of interactions depending on the ligand size and chemistry. For TEA, the phenolic hydroxyl and aromatic ring of Y379 likely participate in cation-π and/or hydrogen-bonding interactions with the quaternary ammonium group of TEA. For HWTX-XI, which is a larger peptide, Y379 could be part of an extended interaction interface—possibly through hydrophobic packing, π-stacking, or side-chain complementarity with toxin residues—that stabilizes toxin–channel binding. Thus, while TEA and HWTX-XI differ greatly in size and structure, Y379 may serve as a versatile “hotspot” that contributes to high-affinity binding in both cases, albeit via distinct interaction mechanisms. Indeed, previous studies have mapped the binding site of several external blockers such as the snake blocker alpha DTX (Figure 8D) [34], the sea anemone blockers BgK (Figure 8F) [29] and ShK [35], and the scorpion blockers Agitoxin 2 [36] and KTX [37] to the conserved tyrosine of the selectivity filter of voltage-gated potassium channels (Figure 8A). In this study, we found that substituting Tyr379 with valine in the filter region reduced the affinity of HWTX-XI by approximately 90-fold. These results supported the inference that the filter region would interact with spider toxin HWTX-XI and played a definitive role in HWTX-XI selectivity.

To elucidate the interaction between HWTX-XI and potassium channels, we compared its three-dimensional structure with that of the Kv1 channel toxin α-dendrotoxin (α-DTX) [38], DTX–K [39] and BgK [22]. The global polypeptide fold of HWTX-XI, alpha DTX and DTX–K is very similar and has an N-terminal 3_10_-helix and a C-terminal α-helix, plus a triple-stranded anti-parallel β-sheet connected by several reversals (Figure 8C–E). A clear trend of enrichment of basic residues is found in key sites which have been shown to form an interacting surface with the Kv1 potassium channel, including R3, R4, K5, L6, I8 and L9 in alpha DTX (Figure 8D). Substitution of Leu9 decreased the affinity of alpha DTX by more than 1000-fold [40]. Similar to alpha DTX, HWTX-XI has a hydrophobic residue Leu at this position. Yuan et al. [28] showed that substitution of Leu with Ala or Tyr led to a ~200-fold reduction in inhibitory potency and therefore plays a critical role in HWTX-XI binding to the Kv1.1 channel. Previous studies indicate that six residues, including K3, K6, W25, K26, and K28, are critical for DTX-K binding to Kv1 channels [41,42]. The mutant K3A decreases the affinity of DTX-K to Kv1.1 by about 1000-fold [42], which is a key residue for channel binding, and is substituted by an Asp residue in HWTX-XI. This mutation might explain the lower affinity of HWTX-XI. Another possible reason for the low blocking potency of HWTX-XI is that the residues corresponding to W25 and K26 of DTX-K, which play an important role in DTX-K binding to the turret of Kv1.1 channels, are missing in HWTX-XI (Figure 1 and Figure 8). BgK is a Kv1 potassium channel blocker from the sea anemone *Bunodosoma granulifera*, which comprises two short helices in its structure [29]. The alanine-scanning mutagenesis study and structures of the complex BgK/S5-S6 region of Kv1.1 indicate that the binding sites of BgK, including the residues F6, S23, K25, and Y26 (Figure 8F), might interact with the residues Y375 and Y379 from Kv1 potassium channels [22,29]. We further elucidated the structural basis for the specific inhibition of the Kv1.1 potassium channel by the neurotoxin HWTX-XI via molecular docking assays. As shown in Figure 8G, HWTX-XI binds to the outer vestibule region of the Kv1.1 channel, and its key functional residues can bind to a characteristic binding pocket on the channel surface. Quantitative analysis of the binding interface (Figure 8H) identified multiple key intermolecular interaction sites. On the toxin molecule, residues L5 and R6 form relatively close interactions with the channel, confirming their core roles; in addition, residue R25 on HWTX-XI also participates in the binding process. On the Kv1.1 channel side, residue Y379 is the most critical interaction partner, with the largest number of contact sites with toxin residues; meanwhile, residues A352 and E353 are confirmed to be important components of the binding site. A magnified view of the interaction interface (Figure 8I) reveals the hydrogen bond forces that maintain the stability of the complex.

Thus, this information implied that K5, L6 and R25 might form an interacting surface to interact with the hydrophobic residues (for example Y379) above the filter region of Kv1 potassium channels (Figure 8). These findings elucidate how a channel blocker discriminates among closely related isoforms, thereby informing the design of analogs with enhanced selectivity.

Kv1.1 mRNA is present in the DRG, and immunocytochemistry confirms that the channel protein is expressed, particularly in small DRG neurons [43]. In addition, Kv1.1 knockout mice display hyperalgesia, implying the important role of these channels in nociception [44]. By balancing the activity of mechanosensitive excitatory channels, a recent study revealed that Kv1.1 channels act as mechanical brakes in the senses of touch and pain and demonstrated that Kv1.1 channels play a central role in mechanical perception [4]. κ-DTX, which is a selective blocker of homomeric and heteromeric Kv1.1 channels, induces repetitive action potential firing of sensory neurons and dramatically decreased paw withdrawal mechanical threshold in mice [4,15]. Because HWTX-XI blocked the pore region of Kv1.1 channels and significantly increased the firing frequency in mouse DRG neurons, we are not surprised that HWTX-XI could induce long-lasting mechanical hyperalgesia in mice (Figure 6). One of the most common functions of the venom in spiders is defense, as spiders use their powerful neurotoxic venom to defend themselves against predators or potential enemies [45]. According to clinical records, the majority of venomous spider *Cyriopagopus schmidti* bites in humans caused apparent local effects, such as local swelling and pain around the bite sites [46]. Ultimately, elucidating the mechanisms underlying venom-induced pain will translate into improved treatments for bite victims and inform the design of new pharmacological agents to alleviate their suffering.

It should be noted that this study has several limitations. First, while molecular docking provided a plausible structural model for the HWTX-XI–Kv1.1 interaction, the inherent uncertainties of computational modeling mean that the proposed binding details require further experimental validation, such as co-crystallography or cryo-EM. Second, our mutagenesis focused primarily on key residues like Y379 and specific toxin interfaces; other channel or toxin regions that may contribute to binding affinity and selectivity warrant systematic exploration. Third, the functional data were obtained mainly from heterologous expression systems and in vitro DRG neuron assays, which may not fully capture the native neuronal environment or in vivo modulatory complexity. Finally, the proposed causal chain from Kv1.1 block to neuronal hyperexcitability and ultimately to mechanical hypersensitivity remains exploratory; further in vivo pathophysiological and behavioral studies are needed to solidify this mechanistic link. Addressing these limitations in future work will strengthen the translational relevance of our findings.

## 4. Conclusions

**Figure 9:**
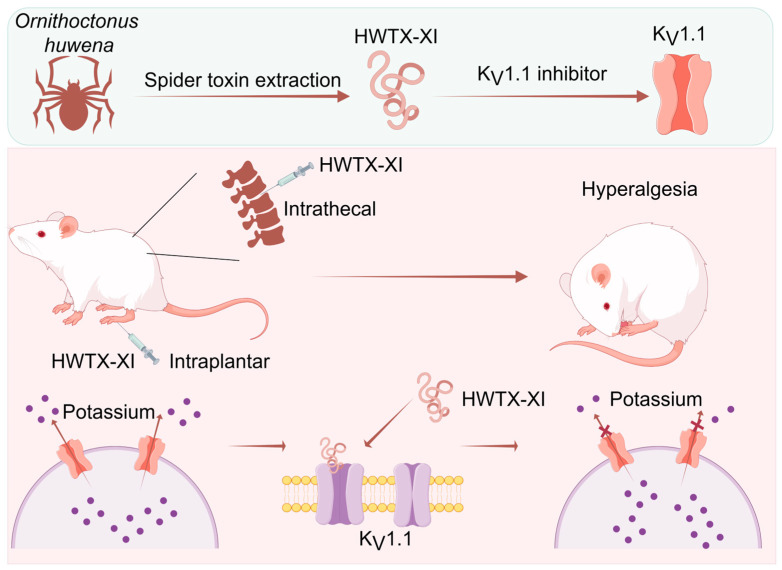
HWTX-XI inhibits the Kv1.1 channel, leading to the development of mechanical sensitivity. Our findings demonstrate that HWTX-XI inhibits the Kv1.1 channel in a concentration-dependent manner by interacting with the channel’s filter region, which not only advances the mechanistic understanding of spider-venom-related pain but also establishes HWTX-XI as a molecular tool and suggests Kv1.1 as a potential target for managing neuropathic pain.

In conclusion, we found that HWTX-XI could block Kv1.1 channels in a concentration-dependent manner by interacting with the S5-S6 region of the channels. This new information advances our understanding of the molecular mechanism involved in the prolonged and intense pain associated with spider envenoming and contributes to establishing an improved pharmacological approach for bitten patients. The novel function of HWTX-XI makes it a new tool for studying the relationship between spider toxins and Kv1.1 channels and suggests that Kv1.1 channels might be a novel potential target for preventing and/or treating neuropathic pain (Figure 9).

## 5. Materials and Methods

### 5.1. Construction and Expression of HWTX-XI

The gene encoding the mature HWTX-XI peptide was subcloned into the expression vector pVT102U using flanking XbaI and Hind III sites. Site-directed mutagenesis was performed by PCR using this construct as a template. The mutated genes were digested with XbaIand HindIII, ligated into pVT102U, and transformed into Saccharomyces cerevisiaestrain S-78 via the LiAc method. Transformants were cultured at 30 °C in YPD medium for 3–4 days. The recombinant peptide HWTX-XI was expressed and secreted into the YPD medium and was detected in the culture supernatant after a 3-day growth period. The 0.45 µm-filtered sample was first subjected to cation-exchange chromatography on a CM-32 column. The collected fractions were subsequently purified by reverse-phase HPLC. The molecular mass of the purified product was verified by MALDI-TOF mass spectrometry.

### 5.2. Plasmids and Construction of Kv1.1 Mutants

The cDNA genes encoding mouse Kv1.1, rat Kv1.2, and human Kv1.3 were subcloned into the vectors pCI, pcDNA3, and pCI-neo, respectively. All mutations of Kv1.1 (A352P, E353T, H355Q, Y379V, A352P/E353T, and A352P/E353T/H355G) were constructed using the GeneTailor™ Site-Directed Mutagenesis kit (Invetrogen, Carlsbad, CA, USA) according to the manufacturer’s instructions. Forward and reverse primers, each 30–34 nucleotides in length, were designed for the procedure, and only one nucleotide was replaced in the A352P (GCT→CCT) and H355Q (CAC→CAG) mutations, with the exception of the E353T, H355G, and Y379V mutations in Kv1.1, where multiple nucleotides were replaced (GAG→ACC, CAC→GGC, and TAC→GTG). All constructs were sequenced to confirm that the appropriate mutations were made.

### 5.3. Transient Transfection

HEK293T cells were maintained in DMEM supplemented with 10% FBS at 37 °C with 5% CO_2_. Cells were transiently transfected with mKv1.1, rKv1.2, or hKv1.3 constructs along with a GFP reporter plasmid using Lipofectamine 2000 (Invitrogen, USA) according to the manufacturer’s instructions. The lipid–DNA complex was incubated with cells for 4–6 h before replacing the medium. Transfected cells exhibiting GFP fluorescence were selected for whole-cell patch-clamp recordings 36–72 h post-transfection.

### 5.4. Dissociation of Mouse Dorsal Root Ganglion (DRG) Neurons

Adult mouse DRG neurons were acutely dissociated and maintained in primary culture, following procedures adapted from Deng et al. [48]. Adult C57BL/6 mice (6–8 weeks old) were anesthetized and decapitated. The thoracic and lumbar vertebral column was removed, and dorsal root ganglia (DRGs) were quickly dissected into ice-cold DMEM. After removing connective tissue under a microscope, the DRGs were minced and digested at 34 °C for 30 min in DMEM containing 0.5 mg/mL trypsin (type III) and 1.0 mg/mL collagenase (type IA). Digestion was terminated with 1.5 mg/mL trypsin inhibitor (type II-S). The cell suspension was centrifuged (800 rpm, 5 min), and the pellet was resuspended in DMEM supplemented with 10% newborn calf serum and 3.7 g/L NaHCO_3_. Cells were plated on 35 mm dishes and maintained at 37 °C in 5% CO_2_ for 1–4 h prior to patch-clamp recording.

### 5.5. Whole-Cell Patch Clamp Experiments

Whole-cell patch-clamp recordings were performed at room temperature (22–25 °C) using an EPC-10 amplifier (HEKA, Lambrecht, Germany). Recording pipettes (2.0–3.0 MΩ) were filled with internal solution containing (in mM): 140 KCl, 1 CaCl_2_, 2.5 MgCl_2_, 10 HEPES, 11 EGTA, and 5 ATP (pH 7.2 with KOH, 310 mOsm/kg H_2_O with sucrose). The bath solution contained (in mM): 150 NaCl, 5 KCl, 2.5 CaCl_2_, 2 MgCl_2_, 10 HEPES, and 10 D-glucose (pH 7.4 with NaOH, 340 mOsm/kg H_2_O with sucrose). For sodium current recordings, the internal solution contained (in mM): 140 CsF, 1 EGTA, 10 NaCl, and 10 HEPES (pH 7.3). The external solution contained (in mM): 140 NaCl, 3 KCl, 1 MgCl_2_, 1 CaCl_2_, and 10 HEPES (pH 7.3). Cells were monitored for 10 min, and those exhibiting significant current rundown were excluded. Series resistance (~5 MΩ) was compensated 65–70%, and linear capacitance/leak currents were subtracted using a P/4 protocol.

### 5.6. Two-Microelectrode Voltage Clamp Experiments

Capped cRNAs encoding ion channels were synthesized after linearizing the plasmids and performing the transcription by a standard protocol [24]. For in vitro transcription, the plasmid pCI containing the Kv2.1 [49] was first linearized with *NotI*; the plasmid PcDNA3.1 containing the gene for Kv4.2 [50] was linearized with SmaI or *Pst I*; the pSP64 plasmids containing the genes for Kv1.4 [51] and Kv3.1 [52] were linearized with *EcoRI*. Using the linearized plasmids as templates, cRNAs were synthesized in vitro using the large-scale SP6 or T7 mMESSAGE mMACHINE transcription kit (Ambion, Austin, TX, USA).

Stage IV–VI oocytes were harvested from anesthetized female *Xenopus laevis* and maintained on ice. Follicle cells were removed by digestion with 1 mg/mL collagenase in calcium-free ND96 solution (in mM: 96 NaCl, 2 KCl, 1 MgCl_2_, 10 HEPES, pH 7.5). Within 2–24 h after defolliculation, oocytes were microinjected with 41 nl of cRNA (100–500 ng/µL) using a microprocessor-controlled injector (WPI, Sarasota, FL, USA). Finally, injected oocytes were incubated at 18 °C for 1–4 days in OR2 solution (in mM: 82.5 NaCl, 2.5 KCl, 1 CaCl_2_, 1 Na_2_HPO_4_, 1 MgCl_2_, 5 HEPES, pH 7.5) supplemented with 50 mg/L gentamycin sulfate.

Whole-cell currents from oocytes were recorded using a two-microelectrode voltage clamp (TURBO TEC-03X, NPI Electronic, Tamm, Germany) at room temperature (19–23 °C). Electrodes (0.1–1 MΩ) were pulled from borosilicate glass and filled with 3 M KCl. During recordings, oocytes were continuously perfused with an extracellular solution containing (in mM): 50 RbCl, 50 NaCl, 1 MgCl_2_, 0.3 CaCl_2_, and 5 HEPES (pH 7.5 with NaOH). Signals were low-pass filtered at 2 kHz and sampled at 0.5 ms intervals; linear capacitive and leak currents were not subtracted.

### 5.7. Nociceptive Behavior Tests

Adult male C57BL/6 mice (20–25 g) were used. All procedures complied with the NIH Guide for the Care and Use of Laboratory Animals and were approved by the Institutional Animal Care and Use Committee of Central South University. Surgery and injections were performed under isoflurane anesthesia, with all efforts to minimize suffering. The nociceptive effect of HWTX-XI was assessed by measuring mechanical hyperalgesia. To evaluate administration route-dependent effects, HWTX-XI (4 µg in 10 µL or 20 µL) was delivered via intrathecal or intraplantar injection, respectively. Control mice received equivalent volumes of physiological saline (0.9% NaCl).

The measurement of mechanical hyperalgesia was carried out using the up-down paradigm as described previously [48]. Mice were acclimatized for at least 30 min in suspended chambers with a mesh floor. Subsequently, the plantar surface of each hind paw was stimulated perpendicularly with a series of calibrated von Frey filaments (Stoelting, Wood Dale, IL, USA). Each filament was applied with enough force to cause bending for 6 s. A brisk paw withdrawal was recorded as a positive response. Following the “up-down” method, the filament strength was adjusted based on the animal’s response, and the mechanical threshold (force with a 50% likelihood of eliciting withdrawal) was calculated.

### 5.8. Data Analysis

Data were acquired and analyzed using Pulse/Pulsefit 8.0 (HEKA, Lambrecht, Germany) and SigmaPlot 9.0 (Systat Software, San Jose, CA, USA). Results are presented as mean ± S.E. (*n* = number of independent experiments). Statistical analyses were conducted in GraphPad Prism 7. Dose–response relationships were fitted to Hill Equation (1):

y = 1 − (1 − *f*_max_)/(1 + ([*x*]/*IC*_50_)^*n_H_*^) (1)

where *x* is the toxin dose, *n_H_* is the Hill coefficient (slope parameter), and *IC*_50_ is the median inhibitory dose causing lethality or block of membrane currents, respectively.

## Data Availability

The original contributions presented in this study are included in the article. Further inquiries can be directed to the corresponding authors.

## Abbreviations

The following abbreviations are used in this manuscript:

DMEM	Dulbecco’s modified Eagle’s medium
DRG	dorsal root ganglion
DTX	dendrotoxin
HPLC	high-pressure liquid chromatography
HWTX	huwentoxin
IC_50_	median inhibitory concentration
KTTs	Kuntiz-type toxins
Kv channel	voltage-gated potassium channel
MALDI-TOF	matrix-assisted laser desorption/ionization time-of-flight
rHWTX-XI	recombinant HWTX-XI
TTX	tetrodotoxin
TTX-R	TTX-resistant
TTX-S	TTX-sensitive
